# Optical performance of progressive addition lenses (PALs) with astigmatic prescription

**DOI:** 10.1038/s41598-021-82697-0

**Published:** 2021-02-04

**Authors:** E. De Lestrange-Anginieur, C. S. Kee

**Affiliations:** 1 School of Optometry, Hong Kong Polytechnic University, Hong Kong, China; 2 Interdisciplinary Division of Biomedical Engineering, Hong Kong Polytechnic University, Hong Kong, SAR China

**Keywords:** Optical physics, Optical techniques

## Abstract

The progressive addition lens (PAL) is a spectacle lens design with progressive refractive power changes across the lens surface to provide sharp vision at different viewing distances for patients with reduced accommodative strength. It has gained in popularity not just for presbyopic patients, but also patients with occupational (office, driving, or digital device) and therapeutic (e.g., myopia control) needs. However, despite the increasing prevalence of astigmatism in adults > 40 years old who rely on PAL correction, no metric is available to reflect the optical variation in PALs with astigmatic prescriptions. Based on recent studies, four novel optical metrics sensitive to variation of refractive power across the lens surface of PALs have been developed. These metrics were used to compare the optical performance of PALs of various prescriptions, designs, and manufacturers. For each lens, the refractive power profile was first measured with a Moire-deflectometry-based instrument.The data was then exported and analyzed using a two-dimensional error map for each of the four metrics. The results revealed significant impacts of astigmatic prescription, providing evidence for the usefulness of these metrics in quantifying the optical performance of PALs for patients with astigmatic prescriptions.

## Introduction

PALs are designed to provide continuous, additional converging powers for eyes that need extra refractive powers to see things sharply at different viewing distances. With the increasing popularity of handheld digital devices, PAL fulfills both functional and cosmetic needs for the growing aging population, in whom accommodative demands cannot be supported through the coordination of eye’s ciliary muscles and the crystalline lens. In the clinical setting, selecting PALs is largely dependent on the patients’ visual demands, such that the design of the PAL’s refractive profile can provide clear distance and near vision within the designated zones^1^. However, the integration of multiple lens curvatures to provide additional powers in PAL inevitably induces unwanted optical blurs, the lower region at both sides of PAL being distorted by varying amounts of astigmatic blur^2^. Such optical blurs degrade patient’s foveal and peripheral vision when the eye looks towards the peripheral area of the lens. A detailed analysis of the aberrations of PALs using wavefront sensing analysis showed a dominant contribution of lower-order aberrations (defocus and astigmatism), with only small amounts of higher-order aberrations^3–5^. To analyze the optical performance of PALs, other optical techniques, such as Moire deflectometry have been applied to characterize the refractive power profile across the lens surface^6^. In addition, measurement of the height profile of the back and front surfaces of PALs was also utilized for ray tracing analysis of the interaction between eye and PALs^7^.

PAL design can be broadly divided into “soft” and “hard” designs, based on the concentration of the distorted area. Soft designs have a smoothly varying optical blur across the lens surface, which is traded off by a narrower area providing clear vision (progressive corridor), whereas hard designs provide a larger area for clear vision at the cost of an abrupt change in refractive powers into the distorted region. Psychophysical studies comparing PAL with single vision lenses have indicated that the narrowing of the progressive corridor caused by optical aberrations tends to reduce the performance of PALs in dynamic visual tasks^8,9^. The importance of the refractive profile of PALs has also been highlighted in several clinical studies comparing patient’s subjective preference with lens design^10–13^. Other studies have shown a strong correlation between various optical quality metrics and visual acuity^14,15^. The hypothesis that patients’ visual performance was sensitive to the optics of PALs was later verified^16^ by correlating optical performance with visual performance measured at different optical zones. Although only local metrics were assessed, a close relationship was found between optical and visual performance, demonstrating that fine-tuning the PAL wavefronts (e.g., in personalized PAL designs) can enhance patients’ visual performance. Given the complex spatial distribution of aberrations across the lens surface of PAL, a comprehensive assessment of optical quality would require a global metric that can reflect the optical quality of the whole lens surface area. In this respect, Sheedy^17,18^ proposed to evaluate the power distribution of PALs by estimating the width of clear “zones” designated for distance, intermediate, and near vision, based on the departure from the target prescription. This method provides a quantitative description of the area that provides clear vision, but the method has not been tested in PALs with astigmatic prescriptions^19^.

The primary aim of this study was to develop quantitative global metrics to examine the influence of astigmatism correction on the optical performance of PALs. The hypothesis is that the optical performance of PALs is quantifiable with metrics sensitive to astigmatic prescription. To test this hypothesis, four metrics varying in complexity were developed and used to compare the optical performance of PALs of different prescriptions, designs, and manufacturers. The results indicate that metrics sensitive to astigmatic prescription can quantitatively differentiate the optical performance of PALs.

## Methods

### PAL samples

To compare the influence of astigmatic axis on lens optics, different designs of PALs from three lens manufacturers (Essilor, Hoya, and Zeiss) with identical addition powers (+ 2.00D), but with cylindrical axis of orthogonal direction (i.e., 90 vs. 180), were tested. Because myopia (or short-/near-sightedness) is increasing in prevalence worldwide, especially in East Asian population^20^), distance prescriptions of PALs were based on the characteristics of refractive error reported in a recent study on the Hong Kong Chinese clinical population^21^: − 4.00 DS, − 4.00 DS/− 2.00 DC × 90, and − 4.00 DS/− 2.00 DC × 180. For each lens manufacturer, two popular PAL models with two different progressive corridors were included (see Table 1). A total of 12 lenses from each lens manufacturer (3 prescriptions × 2 models × 2 corridor lengths) were provided in-kind for measurement. All lenses had a refractive index of 1.6.

**Table 1:** Characteristics of progressive addition lenses measured.

Lens manufacturers	Essilor	Hoya	Zeiss
Fitting cross height (mm)	4	6	6
Corridor length (mm)	14 and 17	11 and 14	10 and 14
Model	1. Varilux Comfort 2. Physio	1. Hoyalux Summit Dynamic 2. Lifestyle V + Harmony	1. Classic PAL LT 2. Precision Plus DVP

### Measurement

**Figure 1:**
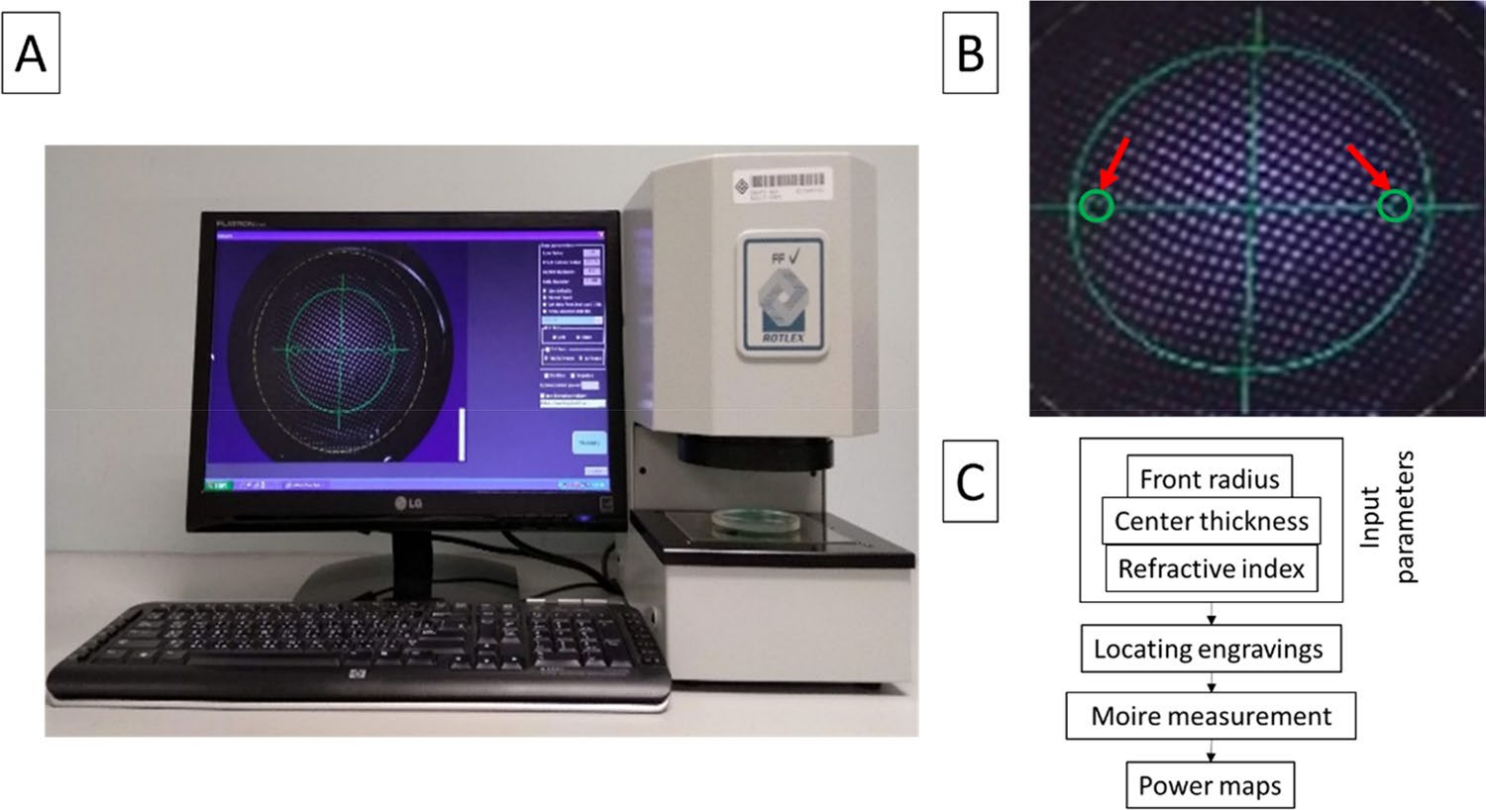
Measurement of refractive power profile. (**A**) The Rotlex Free Form Verifier system with graphic user interface (left) and positioning device (right). Note that the tested PAL was set with the front surface facing downwards. (**B**) The two standard engraved symbols of the PAL marked with a fine-tip marker and positioned within the green rings (arrows) appeared on the graphic user interface. (**C**) The flow chart summarizes the measurement procedure.

Figure 1A–C shows the instruments and steps involved in measuring the refractive power profile of PALs. Based on the Moire deflectometry principle, the Rotlex Free Form Verifier (Rotlex, Omer, Israel) used a diverging light to illuminate the PAL lens surface. Light refracted from the PAL passed through two gratings creating a Moire interference pattern that was converted into arrays of local wavefront properties. These arrays were used to calculate two-dimensional refractive maps of spherical and cylindrical powers. The software required the input of the following lens parameters for the calculation of the refractive power map: lens refractive index (provided by the lens manufacturer), center thickness (measured by a lens gauge), and front surface radius (measured by a lens clock). The locations of the two standard laser-engraved symbols of the PAL were identified with standard procedures and marked by a fine-tip marker. The lens front was placed face down according to the user’s manual. The alignment of the tested lens was checked by positioning the two ink marks (laser-engraved symbols) within the green rings on the screen (Fig. 1B). Once all the parameters were set, five repeated measurements were acquired for each lens and averaged.

**Figure 2:**
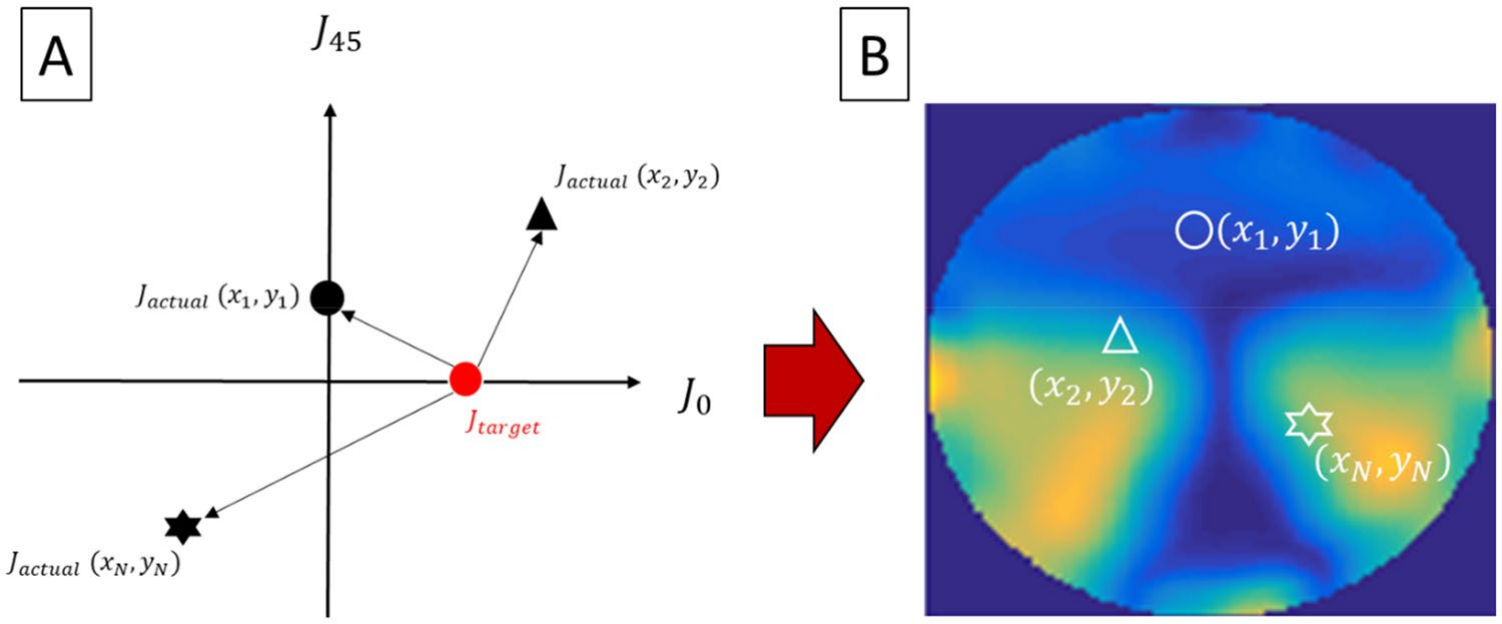
Derivation of cylindrical errors at different locations $\left( {x,y} \right) \,$ on the lens surface. (**A**) Actual cylindrical refractions $J_{actual}$ at three separate locations (black symbols) on the lens surface are represented by their respective J_0_ and J_45_ coordinates. Cylindrical error at each position is quantified by subtracting the target $J_{target}$ (red circle) from the actual $J_{actual}$ (black symbols), as indicated by the arrowhead. (**B**) An error map is constructed by computing the cylindrical error for each location on the lens surface. Cylindrical errors computed for the three locations in (**A**) are represented by open symbols. The color gradient highlights the levels of mismatch between actual and target cylinder power, which progressively increases from blue to red colors.

### Analysis

ASCII data files from the Rotlex Free Form Verifier were imported into Excel and analyzed using a custom Matlab algorithm. The analysis was performed over a 64-mm lens diameter with a resolution of 0.3 mm. Using power vector analysis^22^, the spherical (*S*), cylindrical power (*C*) and axis (*α*) components in the conventional prescription notation ($S, + C \times \alpha + 90$) were converted into rectangular E = (M, $J_{0}$, $J_{45}$) and polar forms (M, $J \times \alpha$):

$$M = S - \frac{C}{2},$$

$$J_{0} = \frac{C}{2}{\text{cos}}\;\left( {2\alpha } \right),$$

$$J_{45} = \frac{C}{2}{\text{sin}}\;\left( {2\alpha } \right),$$

$$J = \sqrt {J_{0}^{2} + J_{45}^{2} } = \frac{C}{2},$$

where M is the spherical equivalent, J_0_, J_45_ and J are the cylindrical components of a Jackson cross-cylinder (JCC) lens at axis 0°, 45°, and $\alpha$, respectively. For instance, for a prescription written in the conventional negative-cylinder notation (− 4.00/− 2.00 × 180), the power vector coordinate is (− 5.00, + 1.00, 0) in rectangular form and (− 5.00, − 1.00 × 90) in polar form.

The formulae above were used to generate a set of optical metrics for comparing refractive power differences, including spherical defocus, astigmatism, and overall optical blurs. To determine the impacts on lens optics due to the varying refractive powers across the lens surface, an error map $\left( \Delta \right)$ of refraction was constructed by subtracting the target refraction from actual refraction of the lens prescription at each position on the PAL surface. The mean and standard deviation of the error maps across the four samples of the same prescription (i.e., 2 models × 2 corridors lengths) are reported for individual manufacturers. To determine the influence of the PAL corridor length and model type on the reported error map, the mean absolute difference of the error maps between PALs having a different corridor length and model, respectively, were also calculated. Small differences indicate that the averaged error map reported is representative of the performance of the selected PAL prescription, whereas large differences indicate that the corridor length or/and model affects the performance of prescription differently.

The error maps generated are described below:

- For unwanted spherical defocus, the error map $\Delta M$ was calculated for three target (desired) viewing distances (i.e., distant, intermediate, and near) of PALs:
  $$\Delta M\left( {x,y} \right) = M_{actual} \left( {x,y} \right) - M_{target} ,$$
  where $M_{actual}$ and $M_{target}$ are the actual (i.e., measured) and target (i.e., intended) spherical powers, respectively. The $M_{target}$ is computed as:
  $$M_{target} = M_{PALs} + V + R_{x} ,$$
  where $M_{PALs}$ were $- 5.00D$ and $- 4.00D$ for PALs with and without astigmatism correction, respectively; V represents the vergences of light (0.00 D, − 1.50 D, − 2.50 D) from three target viewing distances (distant = infinity; intermediate = 66 cm; near = 40 cm); and $R_{x}$ is the residual accommodative power. To account for the residual capacity of an average presbyope to accommodate for nearby objects, as recommended by Andre^23^, a residual accommodative power of $R_{x} = 0.50D$ at the intermediate and near distances was assumed.

- For unwanted astigmatism, two optical metrics, which measure the difference between the actual and target cylindrical profiles at each position on the lens surface, as depicted in Fig. 2, were developed.
- The first metric concentrated on the error between the actual and target astigmatic magnitude of the PALs at each location on the lens surface. The error map associated with a single JCC component at axis $\alpha$, here termed the JCC error map $\Delta J_{\alpha }$, was generated as follows:
  $$\Delta J_{\alpha } \left( {x,y} \right) = (J_{actual} - { }J_{target} ).$$

Although this metric can quantify the difference in magnitudes of actual and target astigmatic blur, it ignores the potential optical effects of astigmatic axis. The JCC error map is equivalent to half of the astigmatic magnitude (“$C$”) in Sheedy’s study^17^.

- To overcome the limitation of $\Delta J_{\alpha }$ of ignoring the astigmatic axis, the second metric, the *vectorial* cylindrical error map, $\Delta J_{v}$ was computed by taking into account the vectorial differences between actual and target astigmatic components of the power vector ($J_{0}$, $J_{45}$), as follows:
  $$\Delta J_{v} \left( {x,y} \right) = \sqrt {\Delta J_{0}^{2} \left( {x,y} \right) + \Delta J_{45}^{2} \left( {x,y} \right)} ,$$
  where the cylinder error maps associated with each $J_{0}$ and $J_{45}$ component were as follows:
  $$\Delta J_{0} \left( {x,y} \right) = J_{0,actual} \left( {x,y} \right) - J_{0,target} ,$$
  $$\Delta J_{45} \left( {x,y} \right) = J_{45,actual} \left( {x,y} \right) - J_{45,target} .$$

**Figure 3:**
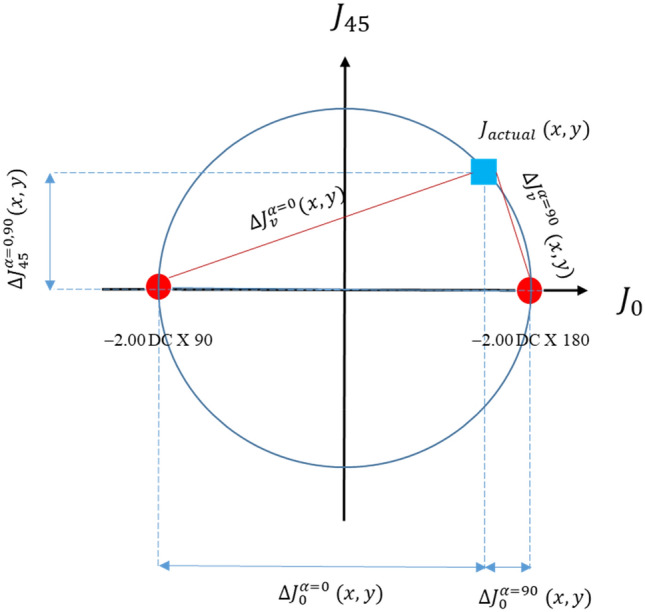
Vectorial differences due to difference in astigmatic axis. Actual (blue square) and target (red circles) refractive prescriptions (− 4.00 DS/− 2.00 DC × 90 and − 4.00 DS/− 2.00 DC × 180) with identical spherical equivalent and cylinder magnitude (*J*) are used as examples. Since all prescriptions have identical spherical equivalent, they lie on the same astigmatic plane. All prescriptions lie on the big blue circle, representing a common cylinder magnitude of $$J$$. The symbol $$\Delta J^{\alpha }$$ stands for the unwanted errors after subtracting the target (with axis $$\alpha = 0$$ and $$90)$$ from the actual prescriptions. Note that in this example, the first metric (JCC error map) predicts the same optical performance for the two prescriptions ($$\Delta J_{\alpha } = 0$$). The second metric (vectorial error map) $$\Delta J_{45}$$ components also could not differentiate between the two prescriptions when compared to the target prescription, but the different $$\Delta J_{0}$$ components for different axes ($$\alpha = 0$$ and $$90)$$ predicted different optical performance (see text for details).

To illustrate how the second metric, $\Delta J_{v}$, can overcome the limitation of the first metric, $\Delta J_{\alpha }$, Fig. 3 shows the vectorial differences that could only be measured by $\Delta J_{v}$ but not the $\Delta J_{\alpha }$ metric. For illustration purposes, the two metrics were compared by their ability to detect differences in astigmatic prescription. Three prescriptions with identical spherical equivalent and cylinder (*J*), one actual (blue square) and two target (red circles) sphero-cylindrical prescriptions, were used for comparison. It should be noted that, because all three prescriptions have identical spherical equivalent (i.e., M =  − 5.00 D), the mean-power dimension (which was represented in the z-axis in Thibos et al. three-dimensional dioptric space^22^) is not shown here. Each of the three symbols occupies a locus on the big blue circle, representing the same magnitude of *J*. Since the first metric ($\Delta J_{\alpha }$) only considers the magnitude of *J*, comparing an actual refractive prescription to either of these two target prescriptions with identical *J* would return a zero value ($\Delta J_{\alpha } = 0)$, i.e., the metric could not differentiate between the two prescriptions with different astigmatic axes. In contrast, $\Delta J_{v}$ takes into account the vectorial differences, returning two distinctly different quantities relative to the actual prescription ($\Delta J^{\alpha }$, represented by red lines), indicating a differential optical performance.

It is important to note that when actual and target axes are the same, the *vectorial* cylindrical error map $\Delta J_{v}$ is equivalent to $\Delta J_{\alpha }$. This is because, for $\alpha_{actual} = \alpha_{target} = \alpha$, the following relationships:

$$\left\{ {\begin{array}{*{20}c} { \Delta J_{0} = J_{actual} cos\; \left( {2\alpha_{actual} } \right) - J_{target} cos \;\left( {2\alpha_{target} } \right)} \\ {\Delta J_{45} = J_{actual} sin\; \left( {2\alpha_{actual} } \right) - J_{target} sin\; \left( {2\alpha_{target} } \right)} \\ \end{array} } \right.$$

become:

$$\left\{ {\begin{array}{*{20}c} { \Delta J_{0}^{2} = \left[ {(J_{actual} - { }J_{target} ){\text{cos}}\; \left( {2\alpha } \right)} \right]^{2} } \\ {\Delta J_{45}^{2} = \left[ {(J_{actual} - { }J_{target} ){\text{sin}}\; \left( {2\alpha } \right)} \right]^{2} } \\ \end{array} } \right.$$

By substituting those expression in $\Delta J$, we get:

$$\Delta J = (J_{actual} - { }J_{target} ) = \Delta J_{\alpha } .$$

- To provide a comprehensive estimation of the overall optical effects introduced by the actual refractive power variation across the lens surface, the length of the power vector^22,24^ was analyzed, as follows:
  $$L = \surd \left( {M^{2} + J_{0}^{2} + J_{45}^{2} } \right).$$

A previous study showed that the length of the power vector can explain more than 90% of the variance in uncorrected visual acuity found in the ametropic population with simple myopia, myopic astigmatism, and mixed astigmatism^15^. In addition, the length of the power vector was found to accurately predict the change in visual acuity associated with optically induced variations in spherical and cylindrical refractive errors, with a correlation coefficient $R^{2}$ ranging from 0.90 to 0.99 in groups with mixed astigmatism, simple myopic astigmatism. and hyperopic astigmatism^25^.

- The overall optical blurs arising from the mismatch between actual and target prescription was computed by vectorial analysis of the power vector according to the above rule of subtraction^26^:
  $$(\Delta M,\Delta J_{0} ,\Delta J_{45} ).$$

The length of this vector difference is a measure of the overall error map of refraction $\Delta L$:

$$\Delta L\left( {x,y} \right) = \surd \left( {\Delta M^{2} \left( {x,y} \right) + \Delta J_{0}^{2} \left( {x,y} \right) + \Delta J_{45}^{2} \left( {x,y} \right)} \right),$$

where $\Delta M, { }\Delta J_{0} ,$ and $\Delta J_{45}$ are the error maps associated with the spherical and cylindrical components, respectively, and are described individually above.

In order to quantify the useful region providing visual clarity on the lens surface, the “area of clear vision” was defined as an area exhibiting dioptric errors below a certain threshold corresponding to the tolerance of optical blur. Here, the area of clear vision within $\pm$ 0.25D of the target prescription was reported, reflecting both the level of accuracy provided by lens prescriptions and the level of blur^17^. This area was used to compare the optical performance between different PALs.

## Results

### Error maps

**Figure 4:**
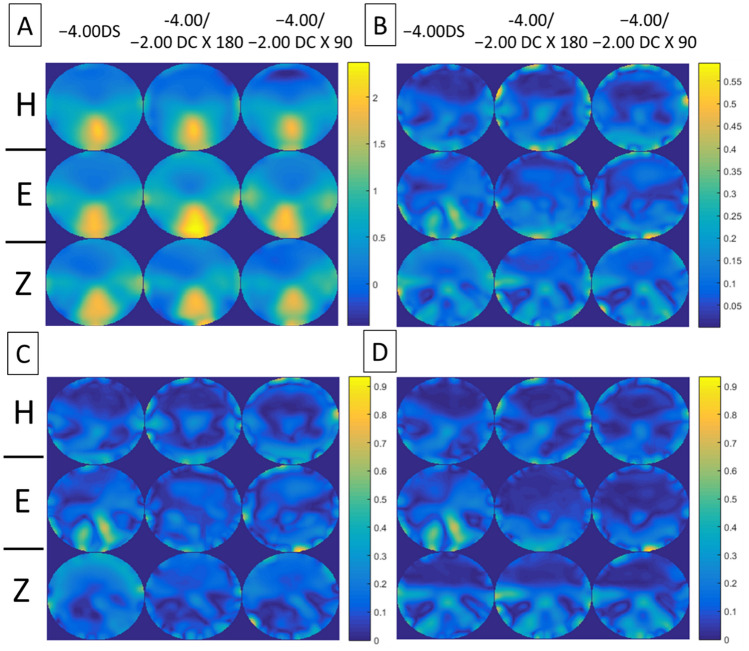
Error maps of spherical power, $\Delta M.$ (**A**) Mean and (**B**) standard deviation of the error maps at distant viewing distance for four lenses (2 models × 2 corridor lengths) with the same prescription (columns) from the three lens manufacturers (rows: *H* Hoya, *E* Essilor, *Z* Zeiss). (**C**) and (**D**) show mean absolute differences of the error maps for PALs of different models and corridor lengths, respectively. Note that the scales of color-coded maps are different.

**Figure 5:**
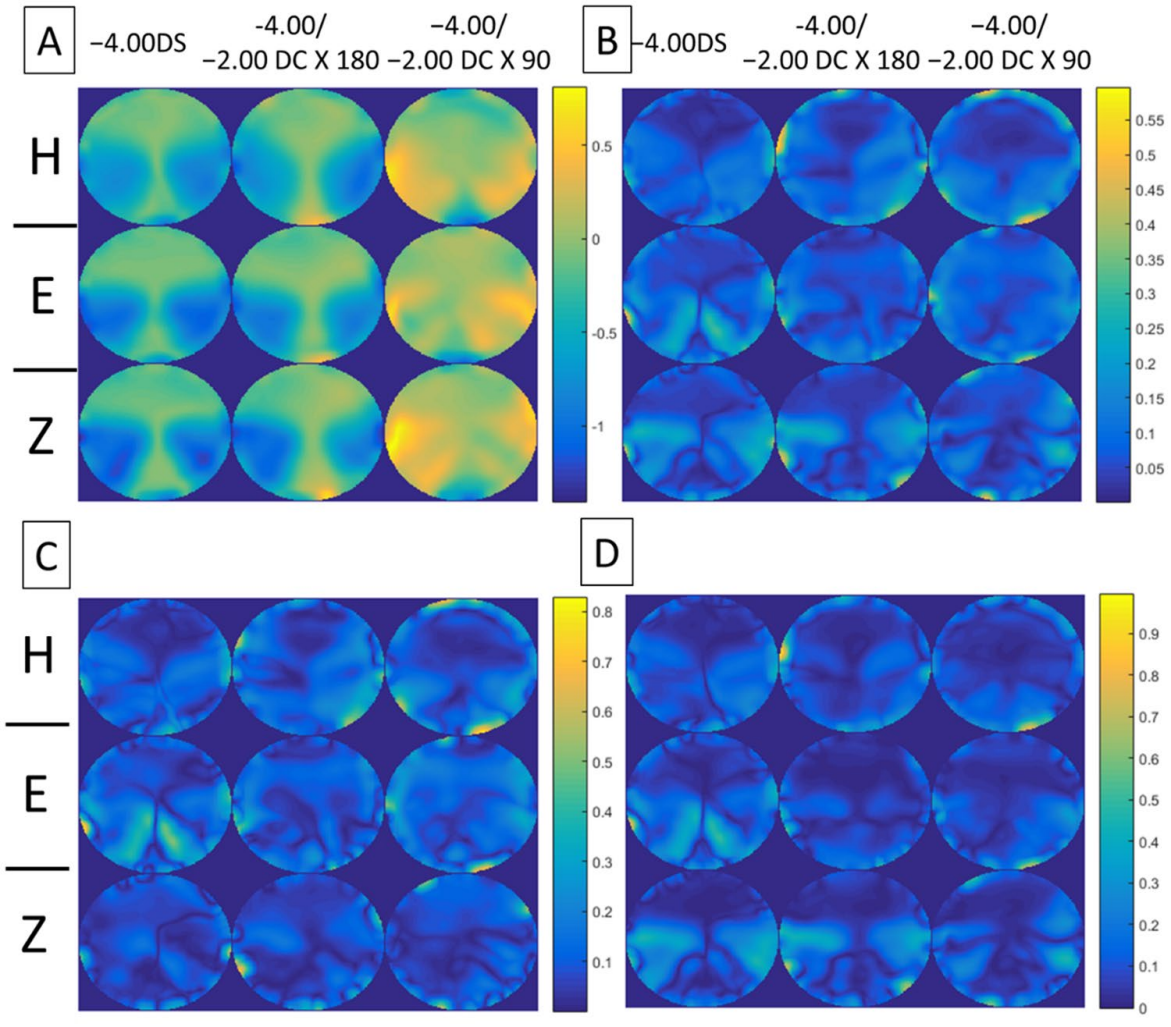
Error maps of JCC, $\Delta J_{\alpha } .$ (**A**) Mean and (**B**) standard deviation of the error maps for four lenses (2 models × 2 corridor lengths) with the same prescription (columns) from the three lens manufacturers (rows: *H* Hoya, *E* Essilor, *Z* Zeiss). (**C**) and (**D**) show mean absolute differences of the error maps for PALs of different models and corridor lengths, respectively. Note that the scales of color-coded maps are different.

**Figure 6:**
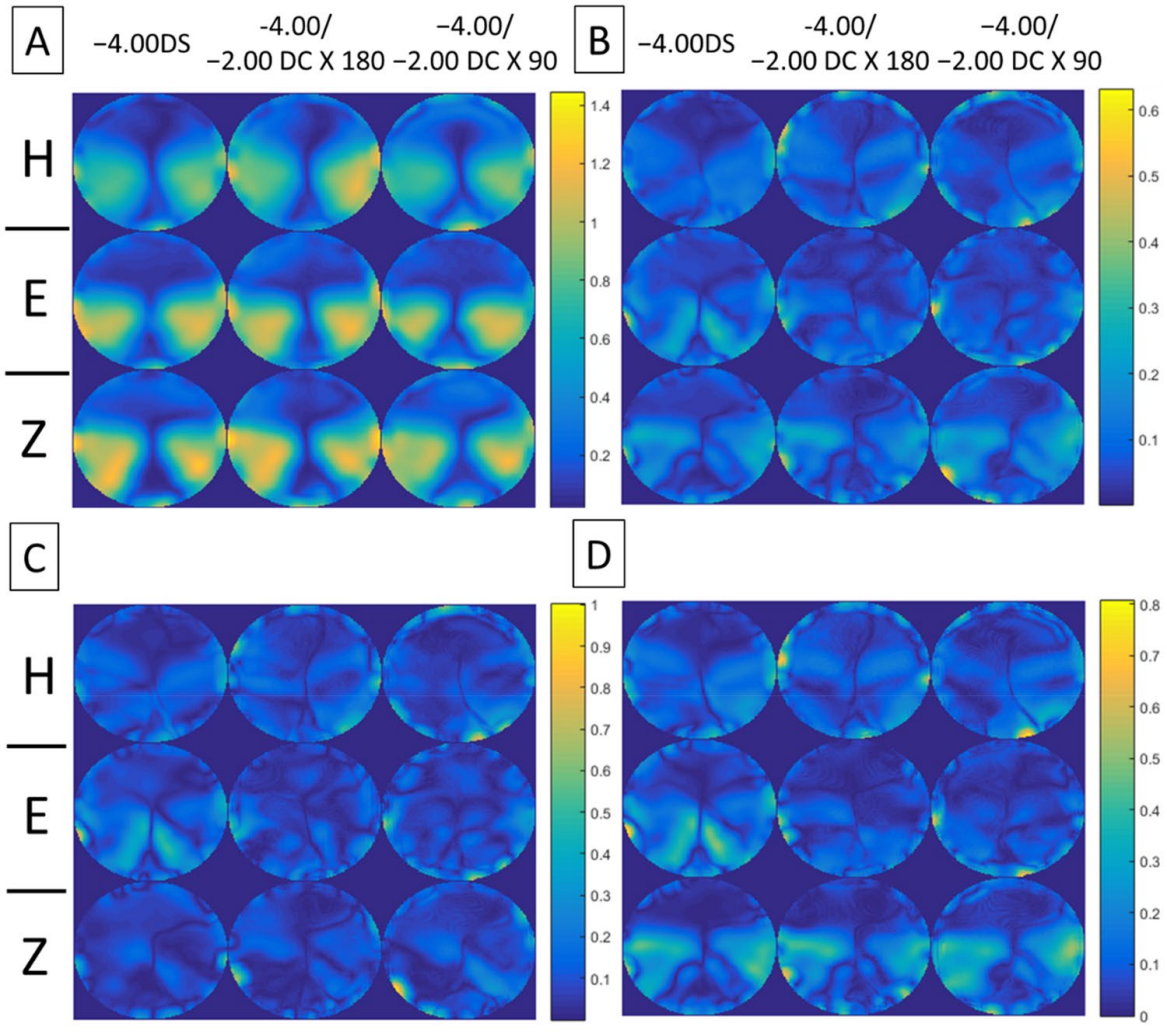
Error maps of vectorial cylinder, $\Delta J_{v} .$ (**A**) Mean and (**B**) standard deviation of the error maps for four lenses (2 models × 2 corridor lengths) with the same prescription (columns) from the three lens manufacturers (rows: *H* Hoya, *E* Essilor, *Z* Zeiss). (**C**) and (**D**) show mean absolute differences of the error maps for PALs of different models and corridor lengths, respectively. Note that the scales of color-coded maps are different.

**Figure 7:**
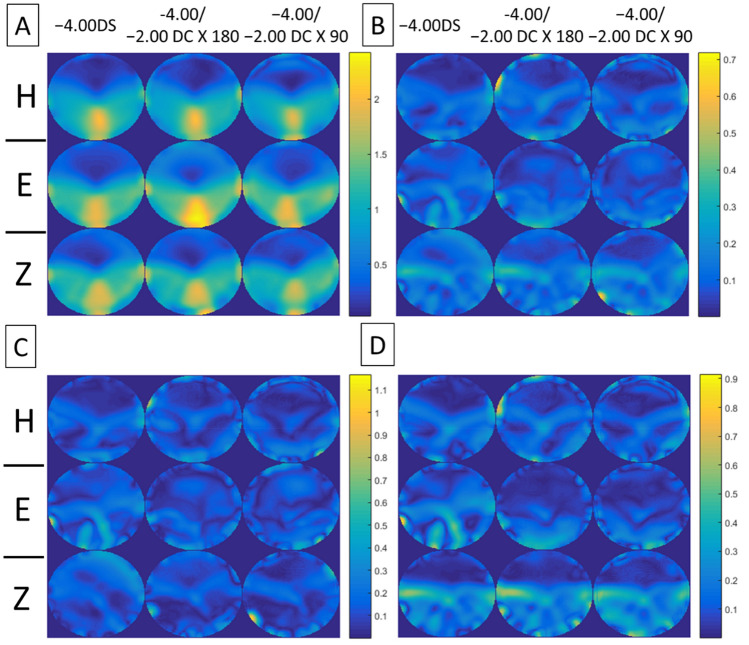
Error maps of overall refraction $\Delta L.$ (**A**) Mean and (**B**) standard deviation of the error maps at distant viewing distance for four lenses (2 models × 2 corridor lengths) with the same prescription (columns) from the three lens manufacturers (rows: *H* Hoya, *E* Essilor, *Z* Zeiss). (**C**) and (**D**) show mean absolute differences of the error maps for PALs of different models and corridor lengths, respectively. Note that the scales of color-coded maps are different.

Figures 4, 5, 6, 7 show the error maps using different optical metrics ($\Delta M$, $\Delta J_{\alpha } , \Delta J_{v}$, $\Delta L$, respectively, defined above) for the three prescriptions (columns) from the three lens manufacturers (rows). In each of these four figures, error maps in (A) and (B) represent the mean and standard deviation of four lenses (2 models × 2 corridor lengths) with the same prescription respectively; whereas maps in (C) and (D) show the absolute differences between samples having different model and corridor length, respectively. Note that the absolute differences in (C) and (D) rarely exceeded 0.40D, indicating that the error maps of the mean in (A) were representative of each PAL prescription. For each prescription (columns), the variation in error maps tended to be similar among manufacturers (A in Figs. 4, 5, 6, 7), indicating similarities in overall design and/or technological approach across the three manufacturers. In contrast, for each manufacturer (rows), qualitative differences in the error maps were observed between the three types of prescriptions, in particular for the JCC error maps ($\Delta J_{\alpha } )$ of PALs with − 2.00 DC × 90 (Fig. 5).

### Area of clear vision

#### $\Delta J_{\alpha }$ metric

**Figure 8:**
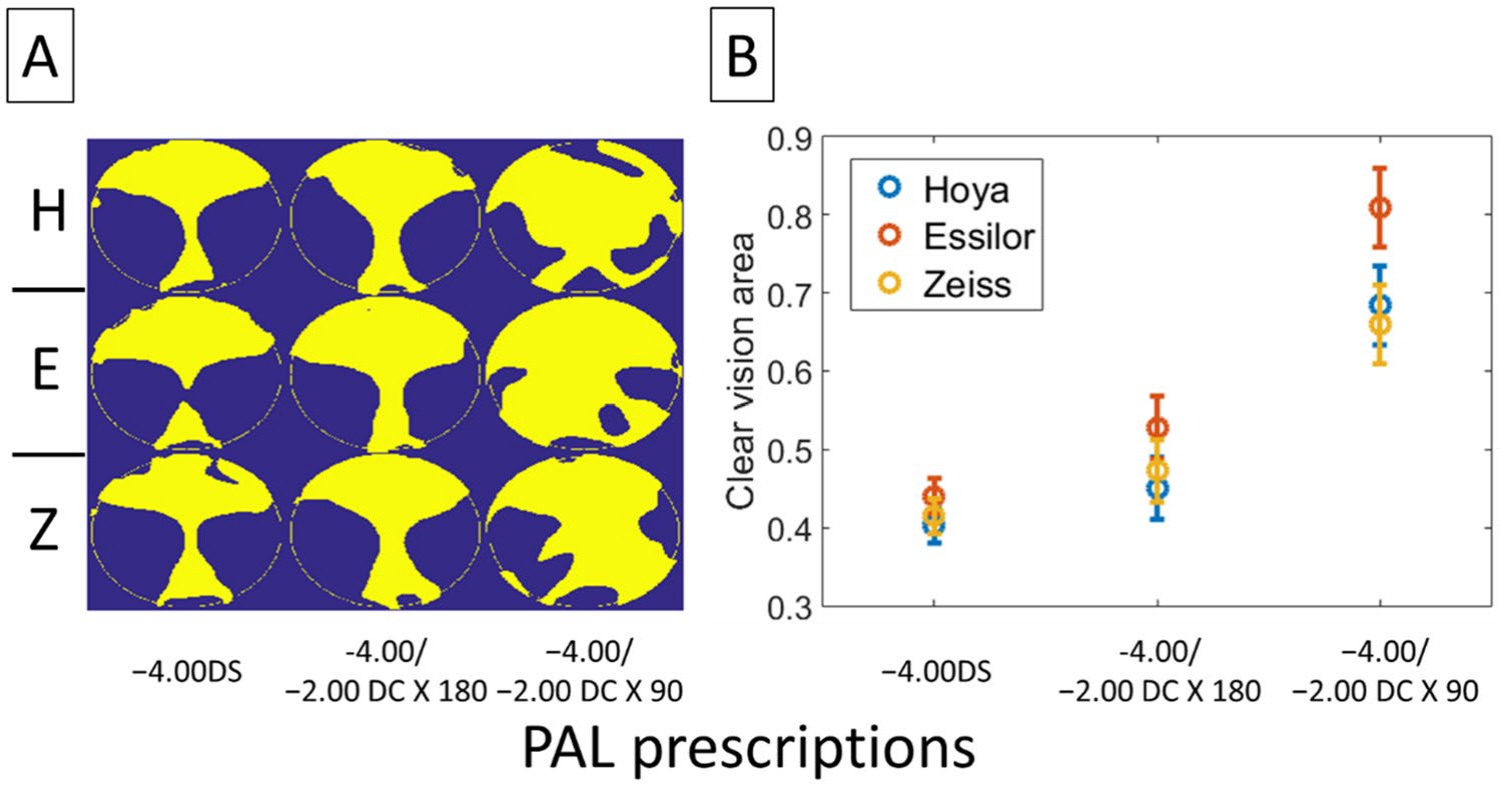
Area of clear vision for different PALs, metric $\Delta J_{\alpha }$. (**A**) Binary image showing area of clear vision (yellow) and area with $\Delta J_{\alpha }$ more than ± 0.25D from the target prescription (dark blue). The calculations in each plot were based on data of four lenses with the same prescription. *H* Hoya, *E* Essilor, *Z* Zeiss. (**B**) Proportion of clear vision area as a function of PAL prescription. The proportion was calculated as the area of clear vision to the total area of measurement. Error bars represent standard errors.

Figure 8 compares the area of clear vision determined by the JCC astigmatic magnitude alone ($J_{\alpha } )$. Using this metric that does not take into account the influence of the astigmatic axis, significant main effects on the area of clear vision were found for both prescription and manufacturer (Two-way ANOVA: prescription effect, p < 0.001; manufacturer effect, p < 0.006). Although all PALs bear some amount of unwanted astigmatism (Fig. 8A, dark blue area), PALs with astigmatic prescription tend to have a smaller magnitude of errors. For instance, the area of clear vision is more than 60% for PALs with − 2.00 DC × 90 prescription (Fig. 8B).

Tables 2 and 3 use colored boxes to indicate significant effects on the area of clear vision for different metrics, as revealed by two-way ANOVA and post-hoc tests, respectively. While an unfilled (blank) box indicates no statistical test is applicable (either because the presence of an interaction effect, or because there was no main effect), the different colored boxes represent different p-values (dark blue, p > 0.05, not significant; light blue, p $\le$ 0.05; yellow, p $\le$ 0.01; red, p $\le$ 0.001). Note that because no interaction effect was found for $\Delta {\varvec{J}}_{{\varvec{\alpha}}}$(dark blue, Table 3), the corresponding column for $\Delta {\varvec{J}}_{{\varvec{\alpha}}}$ in Table 3 has only blank boxes. The following sections cover the results for different metrics, further detailed statistical results are available in Supplementary Tables.

**Table 2:**
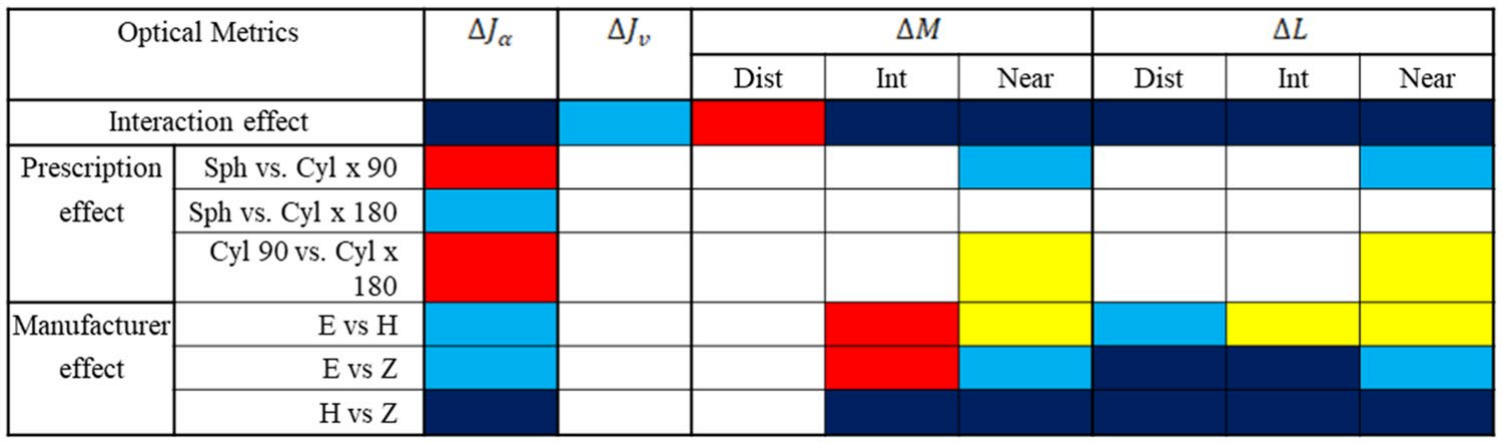
Summary of two-way ANOVA results for different metrics.

This statistical summary indicates the level of significance obtained with various metrics for the different manufacturers and prescriptions. The color code of the p-value is as follows: dark blue, p > 0.05 (not significant); light blue, p $\le$ 0.05; yellow, p $\le$ 0.01; red, p $\le$ 0.001. Note that the blank field corresponds to the conditions where the statistical test is not applicable (i.e., presence of interaction between prescription and manufacturer, or absence of a significant main effect).

*H* Hoya, *E* Essilor, *Z* Zeiss.

**Table 3:**
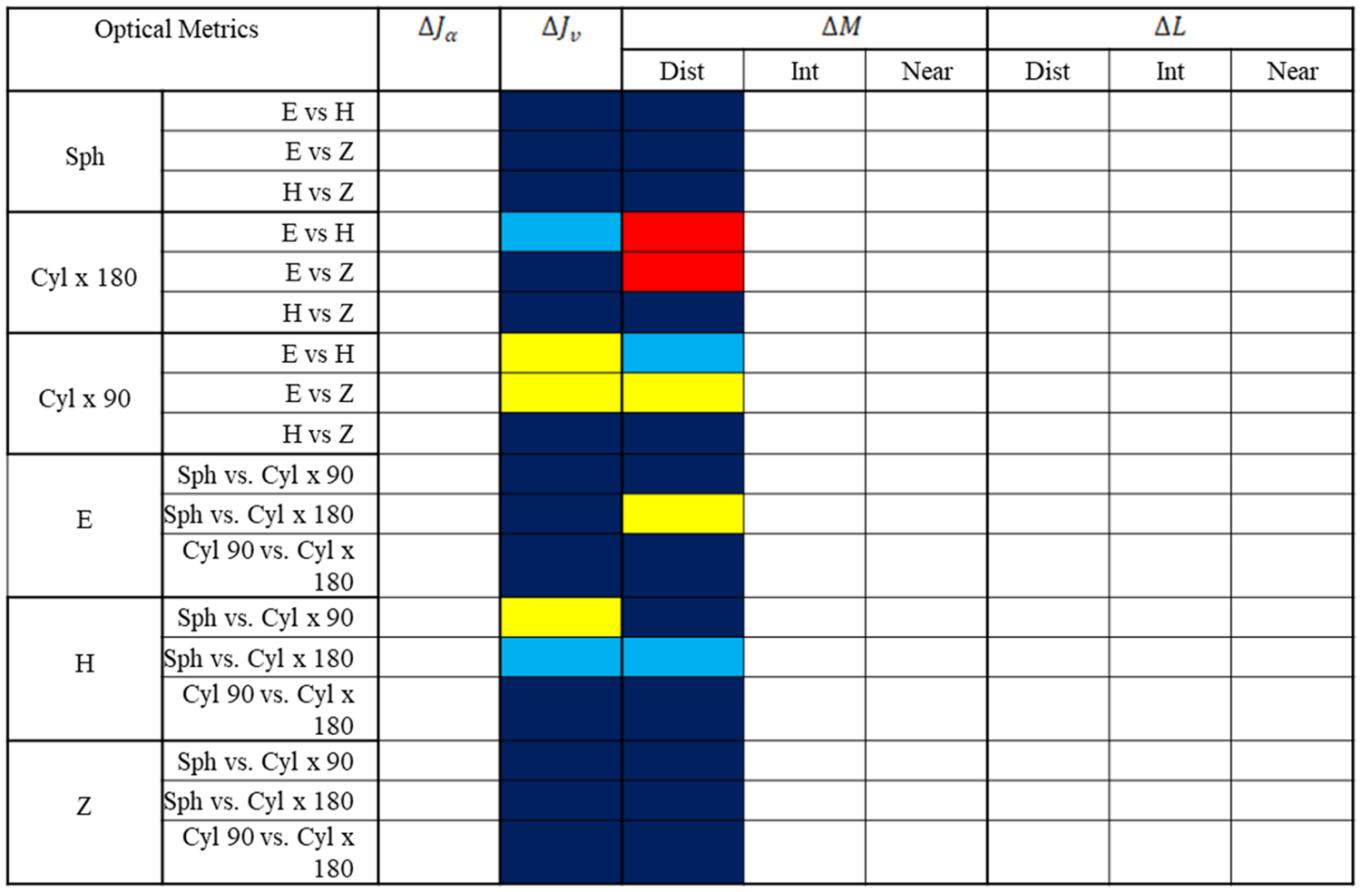
Summary of post-hoc test results for different metrics.

This statistical summary indicates the level of significance obtained with metrics showing interaction between manufacturer and prescription type, namely $\Delta {\varvec{J}}_{{\varvec{v}}}$ and $\Delta {\varvec{M}}$ metric. The color code of the p-value is the following: dark blue, p > 0.05 (not significant); light blue, p $\le$ 0.05; yellow, p $\le$ 0.01; red, p $\le$ 0.001. Note that the blank field corresponds to the conditions where the statistical test is not applicable.

*H* Hoya, *E* Essilor, *Z* Zeiss.

#### $\Delta J_{v}$ metric

**Figure 9:**
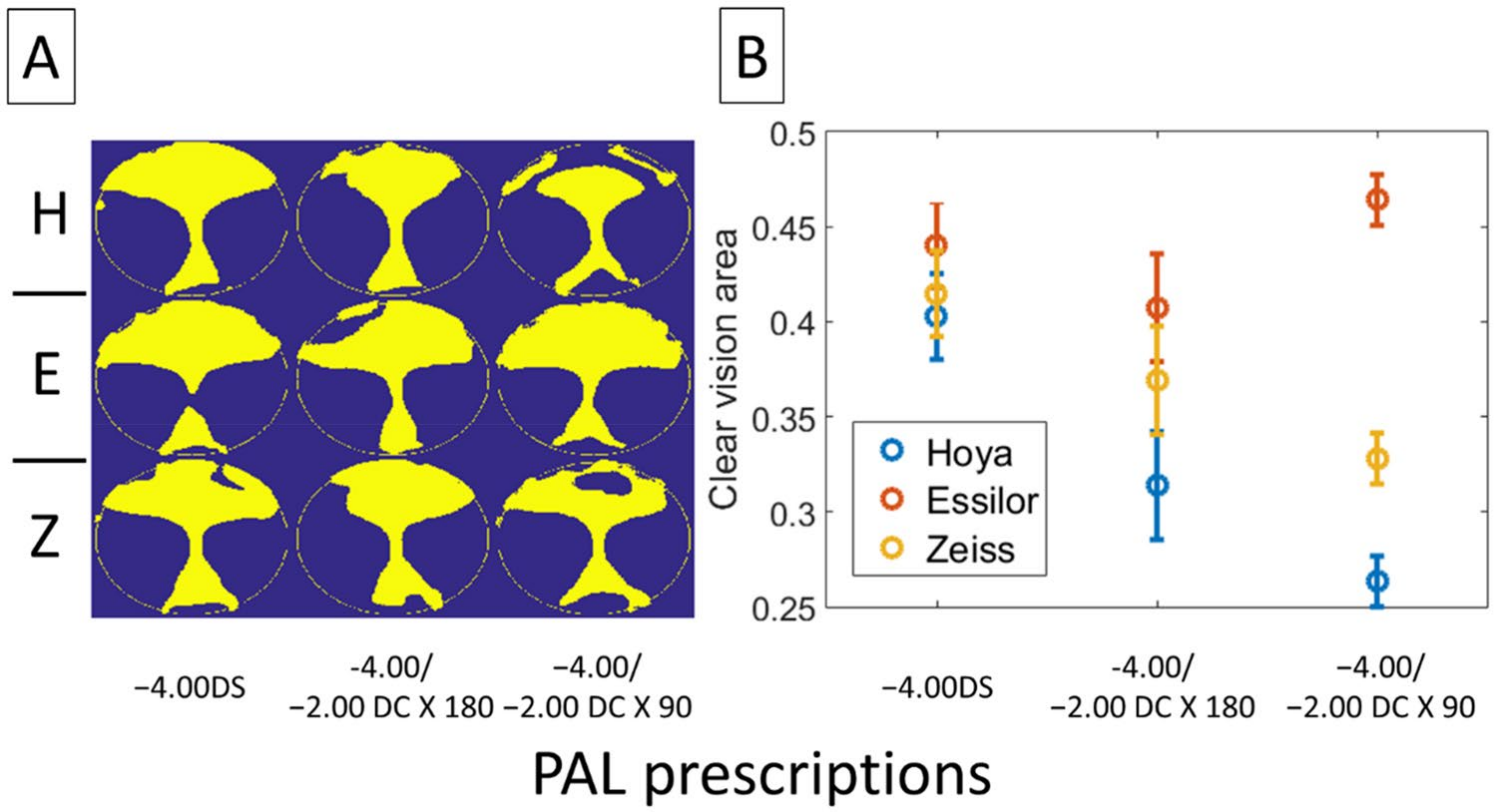
Area of clear vision for different PALs, metric $\Delta J_{v}$. (**A**) Binary image showing area of clear vision (yellow) and area with $\Delta J_{v}$ more than ± 0.25D from the target prescription (blue). The calculations in each plot were based on data of four lenses with the same prescription. *H* Hoya, *E* Essilor, *Z* Zeiss. (**B**) Proportion of clear vision area as a function of PAL prescription. The proportion was calculated as area of clear vision to the total area of measurement. Error bars represent standard errors.

The metric $\Delta J_{v}$, which takes into account the influence of the astigmatic axis, shows a reduction in the range of the clear vision area, as compared to the metric $\Delta J_{\alpha }$(Fig. 9 vs. Fig. 8, 25–45% vs. 40–80% for $\Delta J_{v}$ and $\Delta J_{\alpha }$ metrics, respectively), for PALs with astigmatic correction. These differences were particularly obvious with the − 2.00 DC × 90 prescription, indicating the dominant role of the astigmatic axis in the optical performance. Unlike $\Delta J_{\alpha }$, there was a significant interaction effect when using $\Delta J_{v}$ to compare the area of clear vision across PALs (Two-way ANOVA: manufacturer × prescription, p < 0.05). The difference in optical performance between manufacturers was particularly obvious for PALs with − 2.00 DC × 90 prescription (Fig. 9; see Tables 2 and 3 for statistical details).

#### $\Delta M$ metric

**Figure 10:**
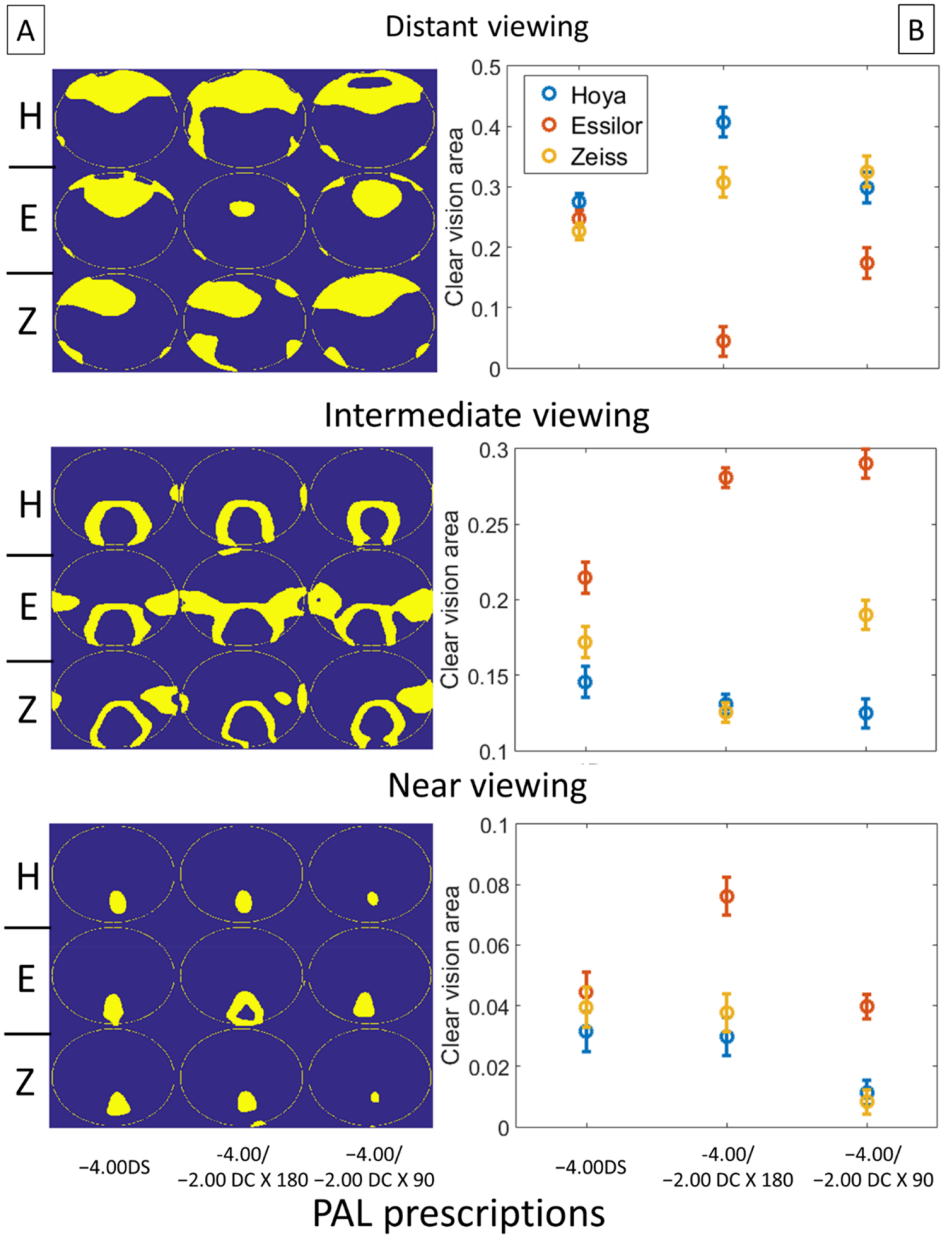
Area of clear vision of different PALs for distant, intermediate, and near viewing distances, metric $\Delta M$. (**A**) Binary image showing area of clear vision (yellow) and area with $\Delta M$ more than ± 0.25D from the target prescription (blue). The calculations in each plot were based on data of four lenses with the same prescription. *H* Hoya, *E* Essilor, *Z* Zeiss. (**B**) Proportion of clear vision area as a function of PAL prescription. The proportion was calculated as area of clear vision to the total area of measurement. Error bars represent standard errors.

The differences in optical performance between PALs were not simply affected by astigmatic magnitude ($\Delta {\varvec{J}}_{{\varvec{\alpha}}}$) and axis ($\Delta {\varvec{J}}_{{\varvec{v}}}$), but tended to vary strongly with viewing distances. Figure 10 shows the area of clear vision determined by the level of spherical error ($\Delta M$) for different viewing distances. At distant viewing distance, there were significant interaction effects of manufacturer and prescription (Two-way ANOVA: p = 0.001); at intermediate viewing distance, only the manufacturer had a significant main effect (p < 0.001); at near viewing distance, both manufacturer and prescription have significant main effects (both p < 0.01) (see Tables 1 and 2 for details). For all the manufacturers, the area of clear vision at near viewing distance was smaller in PALs with − 2.00 DC × 90 prescription, as compared to the other two prescriptions (Two-way ANOVA with post hoc tests; − 2.00 DC × 90 vs. − 2.00 DC × 180, p < 0.01; − 2.00 DC × 90 vs − 4.00DS, p < 0.05).

#### $\Delta L$ metric

**Figure 11:**
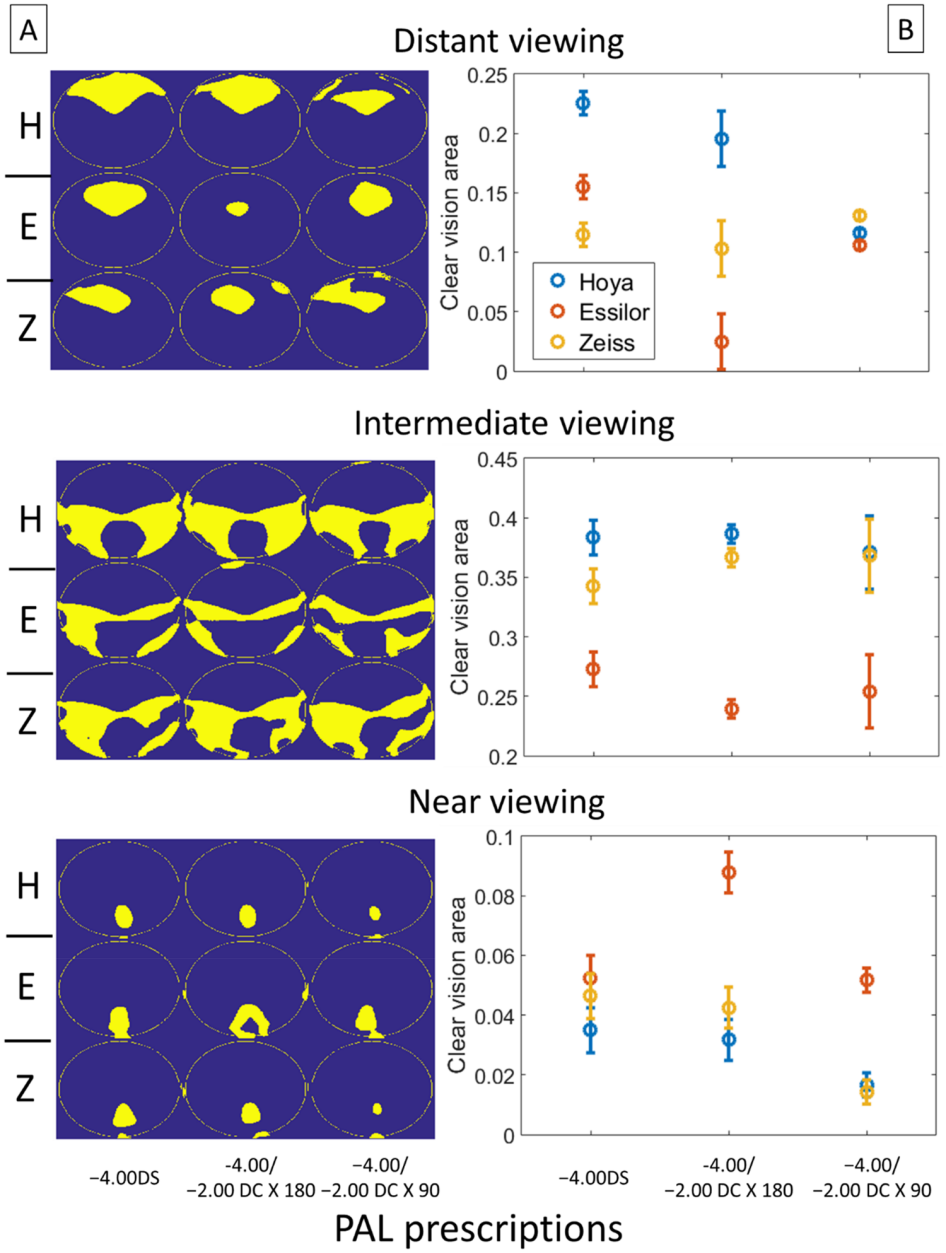
Area of clear vision of different PALs for near, intermediate, and far distance metric $\Delta L$. (**A**) Binary image showing area of clear vision (yellow) and area with $\Delta L$ more than ± 0.25D from the target prescription (blue). The calculations in each plot were based on data of four lenses with the same prescription. *H* Hoya, *E* Essilor, *Z* Zeiss. (**B**) Proportion of clear vision area as a function of PAL prescription. The proportion was calculated as area of clear vision to the total area of measurement. Error bars represent standard errors.

In order to estimate the optical effect due to the combination of spherical errors and astigmatic blurs, Fig. 11 plots the overall errors map of refraction ($\Delta L$) for each viewing distance. For both distant and intermediate viewing distances, the manufacturer has significant main effects (Two-way ANOVAs: p < 0.05 and p < 0.01 respectively); for near viewing distance, both manufacturer and prescription have significant main effects on the area of clear vision (both p < 0.01) (see Tables 2 and 3 for details). Similar to $\Delta {\varvec{M}}$, the area of clear vision using this metric was significantly smaller in PALs with − 2.00 DC × 90 prescription compared to the other two prescriptions (Two-way ANOVA with post hoc tests; − 2.00 DC × 90 vs. − 2.00 DC × 180, p = 0.01; − 2.00 DC × 90 vs − 4.00 DS, p < 0.05).

## Discussion

The four optical metrics developed in this study revealed the impacts of prescription and manufacturer on the optical performance of PALs (Tables 2 and 3). Whilst $\Delta {\varvec{J}}_{{\varvec{\alpha}}} \user2{ }$ metric showed a significant effect of prescription on the optical performance, $\Delta {\varvec{J}}_{{\varvec{v}}} \user2{ }$ metric shows a significant interaction effect of prescription and manufacturer on the area of clear vision (Table 2). In contrast, comparing the optical performance at different viewing distances using $\Delta {\varvec{M}}$ vs. $\Delta {\varvec{L}}$ also revealed subtle differences (Table 2): for distant viewing, the performance depended on the interaction between prescription and manufacturer with $\Delta {\varvec{M}}$**,** but depended only on manufacturer with $\Delta {\varvec{L}}$ (Table 2); for intermediate viewing, it depended on the manufacturer with both $\Delta {\varvec{M}}$ and $\Delta {\varvec{L}}$; for near viewing, it depended on both prescription and manufacturer for both $\Delta {\varvec{M}}$ and $\Delta {\varvec{L}}$. These results highlight the influence of optical parameters on optimizing lens performance across surface area.

The area of clear vision provided by PAL is limited by the axis of the astigmatic correction. Although a metric such as $\Delta {\varvec{J}}_{{\varvec{\alpha}}}$ could reflect the variation of optical blur due to astigmatic *magnitude* (Fig. 8), but the metric per se has little clinical value to one whose vision is sensitive to blur associated with the astigmatic *axis—*in this case a metric such as $\Delta {\varvec{J}}_{{\varvec{v}}}$ provides a better evaluation of optical performance (e.g., compare area of clear vision in Fig. 8 with Fig. 9). Nevertheless, all four metrics including $\Delta {\varvec{J}}_{{\varvec{\alpha}}} \user2{ }$ underscore the pivotal role of astigmatic prescription in determining the optical performance of PALs. Of particular importance is the finding that, as supported by both $\Delta {\varvec{M}}$ and $\Delta {\varvec{L}}$ metrics (Table 2, Figs. 10 and 11), the area of clear vision at near viewing distance shows a significant reduction in PALs with − 2.00 DC × 90 when compared to the other two prescriptions. To the best of our knowledge, this is the first study that shows a significant impact of astigmatic prescription in PALs using novel, quantifiable metrics.

Although these results demonstrate some obvious differences between PALs with different astigmatic correction and between PALs from different manufacturers (Figs. 8, 9, 10, Tables 2 and 3), caution should be employed when generalizing these results to lenses with different prescriptions, because only a small number of PALs were used. Some variations in the distribution of optical errors across PALs are expected with individual lens parameters. In addition to these factors, other non-optical factors can also influence the resultant optical performance. For examples, studies using wavefront analysis^5,16^ showed that the optical interactions between the ocular aberrations and PAL can influence the retinal image quality at specific lens zones. While such optical effects may be overcome by a personalized PAL design taking into account the potential spectacle tilts as-worn (e.g., pantoscopic tilt^4,7^ and wrap angle^11^) and the preferred visual habits (e.g., head and eye movements^9,27^), it remains unknown how the intersubject variability in ocular parameters and the way eye and lens optics interact in real view condition^28^, affect the visual performance of a conventional PAL (i.e. with standard values). Since these parameters may interact with the optics of PALs in a complex manner, prescribing conventional PALs with astigmatic correction for myopic children warrants careful consideration, because growing evidence in animal studies^29^ has indicated the influence of astigmatic blur in regulating early eye growth.

In conclusion, this study used novel quantifiable optical metrics to show the differences in optical performance of PALs attributable to prescription and manufacturer. Further work is needed to investigate the potential impacts of these optical metrics on visual performance in real life conditions.

## Supplementary Information

Supplementary Information.
