# Supplementary material for: Optical performance of progressive addition lenses (PALs) with astigmatic prescription
Source: Sci Rep. 2021 Feb 4;11:2984. doi: 10.1038/s41598-021-82697-0 (PMC7862262; doi:10.1038/s41598-021-82697-0)
Supplement: Supplementary file 1 — Supplementary Information. [file 41598_2021_82697_MOESM1_ESM.docx]

Optical Performance of Progressive Addition Lenses (PALs) with Astigmatic Prescription

De Lestrange-Anginieur E^1*^, and Kee CS^1, 2^

1. School of Optometry, Hong Kong Polytechnic University, Hong Kong
2. Interdisciplinary Division of Biomedical Engineering, Hong Kong Polytechnic University, Hong Kong SAR, China

Supplementary information

Metric $\boldsymbol{\Delta}\boldsymbol{J}_{\boldsymbol{\alpha}}$.

Supplementary Table 1. ANOVA table, metric $\boldsymbol{\Delta}\boldsymbol{J}_{\boldsymbol{\alpha}}$. Two-way ANOVA for manufacturer, prescription type and the interaction. Main significant effects of manufacturer and prescription are found. The symbol † indicates a statistically significant difference (p<0.05).

Supplementary Table 2. Multiple comparison table, metric $\boldsymbol{\Delta}\boldsymbol{J}_{\boldsymbol{\alpha}}$. Bonferroni post-hoc test performed for manufacturer and prescription effects. The symbol † indicates a statistically significant difference (p<0.05).

Metric $\boldsymbol{\Delta}\boldsymbol{J}_{\boldsymbol{v}}$.

Supplementary Table 3. ANOVA table, metric $\boldsymbol{\Delta}\boldsymbol{J}_{\boldsymbol{v}}$. Two-way ANOVA for manufacturer, prescription type and the interaction. A significant interaction between the effect of Manufacturer and Prescription is found, suggesting that the effect of optical performance depends on the interaction of manufacturer and prescription. The symbol † indicates a statistically significant difference (p<0.05).


Supplementary Table 4. Pairwise comparison table for each prescription, metric $\boldsymbol{\Delta}\boldsymbol{J}_{\boldsymbol{v}}$. Bonferroni post hoc test performed for the interaction between manufacturer and prescription. The symbol † indicates a statistically significant difference (p<0.05).

Supplementary Table 5. Pairwise comparison table for each manufacturer, metric $\boldsymbol{\Delta}\boldsymbol{J}_{\boldsymbol{v}}$. Bonferroni post hoc test performed for the interaction between manufacturer and prescription. The symbol † indicates a statistically significant difference (p<0.05).

Metric $\boldsymbol{\Delta M}$.

Supplementary Table 6. ANOVA table, metric $\boldsymbol{\Delta M}$at far distance. Two-way ANOVA for manufacturer, prescription type and the interaction. A significant interaction between the effect of manufacturer and prescription is found. The symbol † indicates a statistically significant difference (p<0.05).

Supplementary Table 7. Pairwise comparison table for each prescription, metric $\boldsymbol{\Delta M}$at far distance. Bonferroni post hoc test performed for the interaction between manufacturer and prescription. The symbol † indicates a statistically significant difference (p<0.05).

Supplementary Table 8. Pairwise comparison table for each manufacturer, metric $\boldsymbol{\Delta M}$at far distance. Bonferroni post hoc test performed for the interaction between manufacturer and prescription. The symbol † indicates a statistically significant difference (p<0.05).

Supplementary Table 9. ANOVA table, metric $\boldsymbol{\Delta M}$at intermediate distance. Two-way ANOVA for manufacturer, prescription type and the interaction. A main significant effect of manufacturer is found. The symbol † indicates a statistically significant difference (p<0.05).

Supplementary Table 10. Multiple comparison table, metric $\boldsymbol{\Delta M}$at intermediate distance. Bonferroni post hoc test performed for manufacturer effects. The symbol † indicates a statistically significant difference (p<0.05).

Supplementary Table 11. ANOVA table, metric $\boldsymbol{\Delta M}$at near distance. Two-way ANOVA for manufacturer, prescription type and the interaction. A main significant effect of manufacturer and prescription is found.

Supplementary Table 12. Multiple comparison table, metric $\boldsymbol{\Delta M}$at near distance. Bonferroni post hoc test performed for manufacturer and prescription effects. The symbol † indicates a statistically significant difference (p<0.05).

Metric $\boldsymbol{\Delta L}$.

Supplementary Table 13. ANOVA table, metric $\boldsymbol{\Delta L}$at far distance. Two-way ANOVA for manufacturer, prescription type and the interaction. A main significant effect of manufacturer only is found.

Supplementary Table 14. Multiple comparison table, metric $\boldsymbol{\Delta L}$at far distance. Bonferroni post hoc test performed for manufacturer effects. The symbol † indicates a statistically significant difference (p<0.05).

Supplementary Table 15. ANOVA table, metric $\boldsymbol{\Delta L}$ at intermediate distance. Two-way ANOVA for manufacturer, prescription type and the interaction. A main significant effect of manufacturer only is found. The symbol † indicates a statistically significant difference (p<0.05).

Supplementary Table 16. Multiple comparison table, metric $\boldsymbol{\Delta L}$at intermediate distance. Bonferroni post hoc test performed for manufacturer effects. The symbol † indicates a statistically significant difference (p<0.05).

Supplementary Table 17. ANOVA table, metric $\boldsymbol{\Delta L}$ at near distance. Two-way ANOVA for manufacturer, prescription type and the interaction. A main significant effect of manufacturer and prescription is found. The symbol † indicates a statistically significant difference (p<0.05).

Supplementary Table 18. Multiple comparison table, metric $\boldsymbol{\Delta L}$at near distance. Bonferroni post hoc test performed for manufacturer and prescription effects. The symbol † indicates a statistically significant difference (p<0.05).
